# Progressive Cerebellar Atrophy Associated with a *KDM5C* Variant

**DOI:** 10.31662/jmaj.2025-0446

**Published:** 2026-07-10

**Authors:** Sato Suzuki-Muromoto, Saki Uneoka, Aritomo Kawashima, Yuki Yagi, Yu Katata, Wakaba Endo, Takehiko Inui, Noriko Togashi, Yusuke Takezawa, Masakiyo Hayasaka, Jun Takayama, Atsuo Kikuchi, Kazuhiro Haginoya

**Affiliations:** 1 Department of Pediatric Neurology, Miyagi Children’s Hospital, Sendai, Japan; 2 Department of Pediatrics, Tohoku University Hospital, Sendai, Japan; 3 Department of AI and Innovative Medicine, Tohoku University Graduate School of Medicine, Sendai, Japan; 4 Tohoku Medical Megabank Organization, Tohoku University, Sendai, Japan; 5 Statistical Genetics Team, RIKEN Center for Advanced Intelligence Project, Tokyo, Japan; 6 Department of Rare Disease Genomics, Tohoku University Graduate School of Medicine, Sendai, Japan

**Keywords:** *KDM5C*, dystonic hypertonia, intellectual disability, cerebellar atrophy

## Introduction

The *KDM5C* gene, located at Xp11.22, is a causative gene of X-linked recessive intellectual disability (XLID), specifically the Claes-Jensen type (MRXSCJ; Online Mendelian Inheritance in Man (OMIM) #300534). Mutations in *KDM5C* account for approximately 1%-3% of all XLID cases ^(1), (2), (3), (4), (5)^. The clinical phenotype associated with *KDM5C* mutations includes a spectrum of features, such as mild to severe intellectual disability, autistic behaviors, epilepsy, spastic paraplegia, hyperreflexia, emotional dysregulation, facial dysmorphism, microcephaly, and short stature ^(1), (2), (3), (4), (5)^. Despite these findings, genotype-phenotype correlations remain poorly understood ^(6)^. Moreover, neuroimaging findings in individuals with *KDM5C* mutations have been infrequently reported. Here, we present an individual with a de novo *KDM5C* variant who exhibited progressive cerebellar atrophy.

## Case Report

The patient was a 9-year-old boy born to non-consanguineous Japanese parents. He was delivered at 34 weeks of gestation due to maternal gestational hypertension. At birth, his weight, length, and occipitofrontal head circumference (OFC) were 1,640 g (−3.2 standard deviation [SD]), 43.0 cm (−2.9 SD), and 30.5 cm (−2.0 SD), respectively. His postnatal course was uneventful, and he was discharged on day 35 of life. Head control was achieved at 5 months of age. At that time, poor eye contact was observed, and he was diagnosed with mild congenital bilateral cataracts. Due to delayed motor development, he was referred to our hospital at 11 months of age. Upon examination at 1 year and 2 months of age, his body weight, length, and OFC were 9.1 kg (−0.1 SD), 72 cm (−1.0 SD), and 44.5 cm (−1.5 SD), respectively. Although he was able to roll over with effort, sit unaided, and play with toys using his hands, his movements appeared clumsy. He demonstrated eye contact, visual pursuit, and social smiling; however, he had not yet developed babbling or spoken words. Mild feeding difficulties were present. At rest, no signs of spasticity or abnormalities were observed in deep tendon or pathological reflexes. However, dystonic hypertonia of the extremities was evident during voluntary movements, characterized by extra-segmental co-contraction of flexor and extensor muscle groups. No apparent facial dysmorphism was observed. Brain magnetic resonance imaging (MRI) at 11 months of age demonstrated delayed myelination. He achieved independent walking at 2 years and 6 months of age, accompanied by the gradual resolution of the dystonic hypertonia. To investigate the etiology, we performed trio-based whole-exome sequencing, as described previously ^(7)^. His family provided informed consent for next-generation sequencing and publication of this case report. The study was approved by the ethics review boards of Miyagi Children’s Hospital and Tohoku University Hospital. We identified a de novo *KDM5C* variant: NM_004187.5 c.1439C>T; p.(Pro480Leu). This variant was classified as pathogenic based on the American College of Medical Genetics and Genomics guidelines (PS1+PS2+PM1+PM2+PP3+PP5). Segregation analysis via Sanger sequencing confirmed the de novo status. No additional pathogenic variants were identified (Table S1). Furthermore, repeat expansion mutations associated with spinocerebellar ataxia types 1, 2, 3, 6, 7, 8, 12, 17, and 31, as well as DRPLA, were excluded.

**Figure 1.**
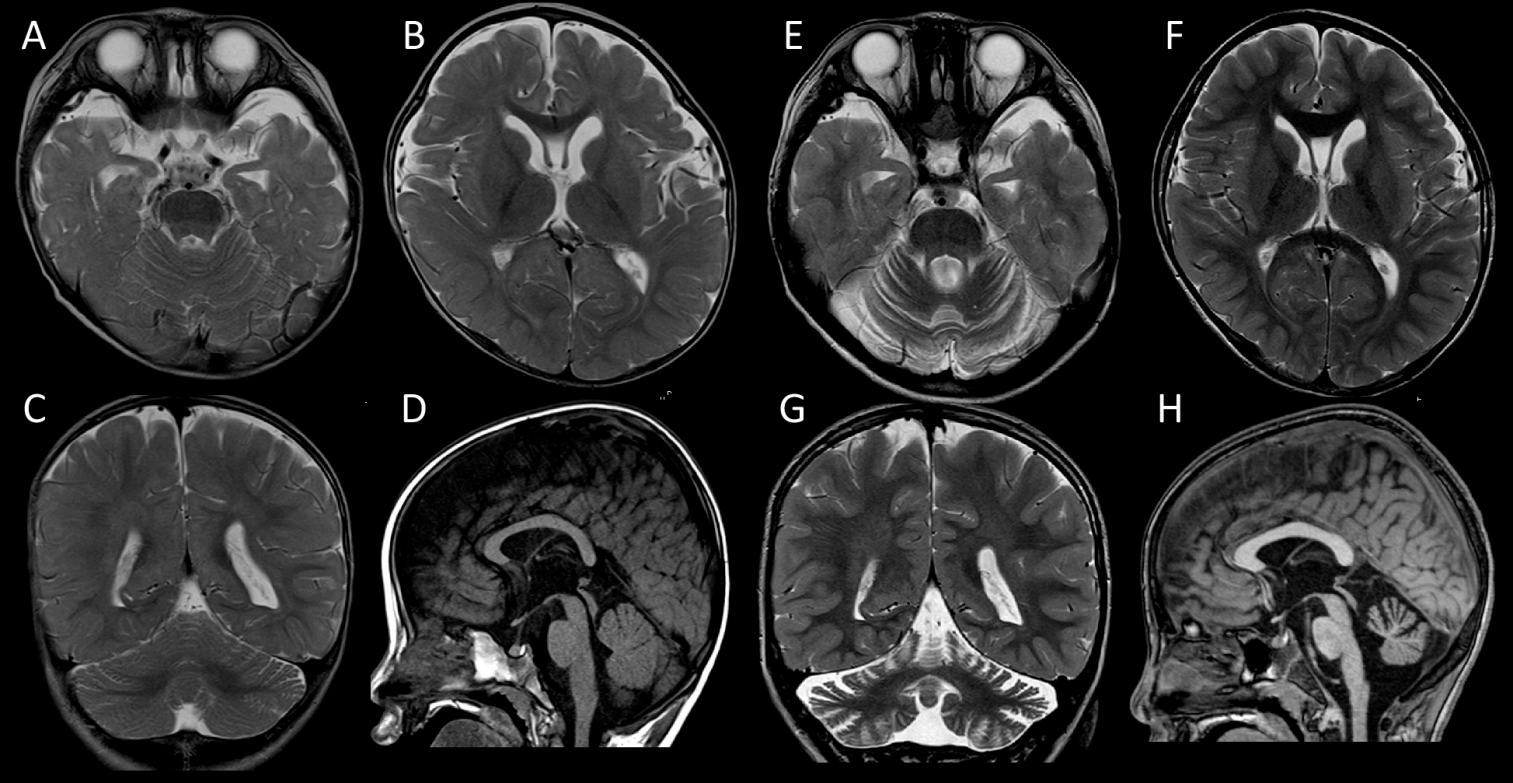
MRI of the brain at 2 years (A-D) and 7 years old (E-H) with axial T2WI (A, B, E, F). Coronal T2WI (C, G) and sagittal T1WI (D, H). MRI at 2 years old, demonstrating persistent delayed myelination. However, by 7 years of age, cerebellar hemisphere and vermis atrophy became apparent, despite the absence of clinical signs of ataxia. Cerebral myelination appeared normal at that time. MRI: magnetic resonance imaging.

He began experiencing tonic seizures at 5 years of age. Electroencephalography revealed frequent spikes and sharp waves arising independently in the bilateral central regions. He was initiated on the antiseizure medication levetiracetam, which resulted in good seizure control. A repeat brain MRI at 2 years of age demonstrated persistent delayed myelination. By 7 years of age, cerebellar hemisphere and vermis atrophy became apparent, despite the absence of clinical signs of ataxia (Figure 1). Cerebral myelination appeared normal at that time.

Currently, he presents with profound intellectual disability without meaningful words, mild short stature, mild bilateral cataracts, mild hypertonia, transient tremulous movements of the extremities, and well-controlled epilepsy. His body weight, height, and OFC are 28.4 kg (−0.26 SD), 124.3 cm (−1.36 SD), and 52.5 cm (+0.1 SD), respectively.

## Discussion

The* KDM5C* gene encodes a transcription factor that contains multiple deoxyribonucleic acid-binding motifs and exhibits histone demethylase activity specific for di- and trimethylated lysine 4 of histone H3 ^(1), (5), (8), (9)^. This enzymatic activity implies a central role for KDM5C in transcriptional repression, contributing to chromatin remodeling and epigenetic regulation during cellular growth, differentiation, and development ^(10)^. Most functionally characterized missense variants in *KDM5C* result in reduced demethylase activity, indicating that loss of function is the predominant pathogenic mechanism ^(1), (11)^. KDM5C is broadly expressed, with particularly high expression in the human brain and skeletal muscle ^(12)^. *Kmdc5*-knockout mice exhibit abnormal dendritic arborization, spine anomalies, and altered transcriptomes, indicating that while *kmdc5* is not essential for gross brain morphogenesis, it plays crucial roles in neuronal development and function ^(9)^.

Brain MRI has been performed in 21 families with *KDM5C* variants, most of which showed nonspecific findings. However, cerebellar atrophy has been reported in three families, all of whom harbored frameshift variants: p.Ala1292Glnfs*7 ^(13)^, p.Thr270Glnfs*2 (VCV000635109.5) ^(14)^, and p.Arg212Glyfs*22/p.Arg211Glyfs*23 ^(15)^. Cerebellar atrophy was identified at 4, 7, 4, and 4 years old, respectively, and at 7 years old in our case (Table S2). Hypertonia of the upper extremities or dystonic hypertonus may represent a unique clinical feature in these individuals. Clear symptoms of cerebellar ataxia have not been reported. On the other hand, because the cerebellum is involved in language, working memory, and social and emotional processing ^(16)^, intellectual disability may be partly explained by cerebellar atrophy. Although the missense variant p.Pro480Leu has been shown to cause a marked reduction in demethylase activity and protein stability ^(1)^, MRI data for this variant have not been reported (Table S3) ^(17)^. Further follow-up is needed to define the full clinical picture in affected individuals, including the present case. In mice, *kmdc5* mRNA is expressed in the hippocampus, hypothalamus, and cerebellum ^(12)^. Considering the loss-of-function profile of this variant, which resembles that of frameshift mutations, it is plausible that *kmdc5* deficiency leads to cerebellar atrophy due to widespread transcriptional dysregulation.

In conclusion, X-linked intellectual disability associated with cerebellar atrophy may represent a phenotypic marker of *KDM5C* variants, particularly in cases involving severe loss of function. However, additional neuroimaging data are required to substantiate this association.

## Article Information

### Acknowledgments

We thank the individual and the individual’s family for their participation in this study. Drs. Takayama and Kikuchi have received a research grant from Astellas Pharma. We also thank Yoko Chiba and Kumi Ito for providing technical assistance.

### Author Contributions

Responsible for the content of this paper: Kazuhiro Haginoya; Contributed gene analysis and interpretation of genetic analysis data: Yusuke Takezawa, Masakiyo Hayasaka, Jun Takayama, Atsuo Kikuchi; Study conceptualization, design, and manuscript preparation: Sato Suzuki-Muromoto; Gathered clinical data and participated in the treatment: Saki Uneoka, Atsuo Kikuchi, Yuki Yagi, Yu Katata, Wakaba Endo, Takehiko Inui, and Noriko Togashi.

### Conflicts of Interest

None

## Supplement

Supplementary Material
