# Supplementary material for: Progressive Cerebellar Atrophy Associated with a KDM5C Variant
Source: JMA J. 2026 Jul 10;9(4):1008–11. doi: 10.31662/jmaj.2025-0446 (PMC13453517; doi:10.31662/jmaj.2025-0446)
Supplement: Supplementary file 1 [file 2433-3298-9-4-1008-s001.pdf]

Supplementary Table 1. Gene Mutations Causing Cerebellar Atrophy Excluded in the Present Analysis

AD	*CACNA1A, ITPR1, KIF1A, APTX, KCNC3, ARF3, ATP1A3, ATP6V1A, CACNA1G, CAMK2B, CTBP1, SCN8A, SPTBN2, PRKCG, NOP56, AFG3L2, KCNC3, FGF14, ELOVL5, KCNA1*
AR	*COQ2, ADCK3, SETX, APTX, ACO2, FXN, ATM, PNPLA6, SYNE1, TTPA, POLG, PLA2G6, VLDLR, RELN, ARG1, FOLR1, GEMIN5, MCOLN1, IHPRF1, PEX16, SIL1, SLC9A1, SNX14,*
X-linked	*CASK, L1CAM, OPHN1, THOC2*

Supplementary Table 2. Clinical Pictures of Patients with *KDM5C* Variants and Cerebellar Atrophy

	present case	Kawano-Matsuda(ref. 13)	Anvari (ref. 14)	Fujita III-2 (ref. 15)	Fujita III-3 (ref. 15)	Fujita III-4 (ref. 15)
					younger brother of III-2	youngest brother of III-2
gene variant	p.Pro480Leu	p.Ala1292Glnfs*7	p.Thr270Glnfs*2	p.Arg212Glyfs*22	p.Arg211Glyfs*23	p.Arg212Glyfs*22
age at onset and initial symptoms	11m, developmental delay, dystonic hypertonia of upper extremities	4y6m, short stature, developmental delay	age, ND, developmental delay	18m, growth and developmenatal delay	3y, seizures, developmental delay	0m, feeding difficulty, 1y, developmental delay
age and findings at first MRI	11m, delayed mylination	4y, cerebellar atrophy	7y, cerebellar atrophy	3y, normal	4y, cerebellar atrophy	1y11m, normal
age and findings at repeat MRI	2y, delayed myalination			4y, cerebellar atrophy		
	7y, cerebellar atrophy			6y, cerebellar atrophy		
				8y, cerebellar atrophy		
symptoms	short stature, developmental delay, epilepsy, dystonic hypertonia of upper extremities in infancy, intellectual disability (severe)	short stature, developmental delay	convulsion, speech disturbance, global developmental delay, restlessness	developmental delay, upper limb rigidity, spasticity in the lower limbs, epilepsy, subclinical hypothyroidism, short stature	developmental delay, increased tonus of upper limbs, subclinical hypothyroidism, short stature	developmental delay, increased tonus of upper limbs, subclinical hypothyroidism, short stature

Abbreviations: ND, not described; y, year; m, month

Supplementary Table 3. Clinical Picture of Patients with p.Pro480Leu of *KDM5C* Gene

	present case	Grafodatskaya (ref. 17)	Grafodatskaya (ref. 17)
gene variant	p.Pro480Leu	p.Pro480Leu	p.Pro480Leu
age at onset and initial symptoms	11m, developmental delay, dystonic hypertonia of upper extremities	ND	ND
age at clinical examination	11m	7y	2y (younger brother)
symptoms	short stature, intellectual disability (severe), epilepsy, dystonic hypertonia of upper extremities in infancy	intellectual disability (moderate), short stature	intellectual disability (mild), short stature, seizures

Abbreviations: ND, not described; y, year; m, month
